
JOURNAL INFORMATION
==============================
NLM Title Abbreviation: ScientificWorldJournal
Journal ID: ScientificWorldJournal
NLM Journal ID: 101131163
Journal ID: TSWJ

The Scientific World Journal

ISSN: 2356-6140
EISSN: 1537-744X
Publisher: Wiley

ARTICLE INFORMATION
==============================
PMCID: PMC6009278
PMID: 29973846
DOI: 10.1100/tsw.2002.71
Article ID: 259840
Article version: 1
Subjects: Short Report

High-Throughput Protein Expression and Purification for Proteomics Research

Doyle Sharon A. *
Murphy Michael
Primus Jennifer
Richardson Paul
Hawkins Trevor
Functional Genomics Division, DOE Joint Genome Institute, 2800 Mitchell Drive, Walnut Creek, CA 94598, USA
*Sharon A. Doyle: sadoyle@lbl.gov
Publication date: 2002
Electronic publication date: 2002 May 21
Volume: 2
First page: 146
Last page: 147
Copyright: Copyright © 2002 Sharon A. Doyle et al.
Copyright year: 2002
License: This is an open access article distributed under the Creative Commons Attribution License, which permits unrestricted use, distribution, and reproduction in any medium, provided the original work is properly cited.
License URL: https://creativecommons.org/licenses/by/3.0/

Funding: Lawrence Livermore National Laboratory, Office of Science W-7405-Eng-48 Lawrence Livermore National Laboratory, Office of Science DE-AC03-76SF00098 Los Alamos National Laboratory W-7405-ENG-36

==============================
Advances in Gene Technology: The Genome and Beyond –
Structural Biology for Medicine (Proceedings of the 2002 Miami
*Nature Biotechnology* Winter Symposium)
*TheScientificWorld* 2002, 2(S2), 146–147
ISSN 1532-2246; DOI 10.1100/tsw.2002.71

### HIGH-THROUGHPUT PROTEIN EXPRESSION AND PURIFICATION FOR PROTEOMICS RESEARCH

Sharon A. Doyle*, Michael Murphy, Jennifer Primus, Paul Richardson, and Trevor Hawkins
Functional Genomics Division, DOE Joint Genome Institute, 2800 Mitchell Drive, Walnut Creek, CA 94598
*sadoyle@lbl.gov

**INTRODUCTION**. Full insight into the control of genomic sequences over many biological processes requires the analysis of the protein products. Only through the analysis of proteins on a genomic scale can we begin to understand the complexities encoded in the genome. Methods that allow for the production of proteins in a high-throughput manner are vital to achieve this goal. We have developed a system for high-throughput subcloning, protein expression and purification that is simple, fast, and inexpensive. We utilize ligation-independent subcloning to create an expression vector encoding a N-terminal histidine tag. A dot blot expression screen was developed to analyze protein levels following expression in bacterial cultures, which facilitates the testing of multiple expression parameters if necessary. Protein purification in a 96-well format using Ni-NTA resin yields highly purified proteins.

**METHODS**. An expression vector was constructed using pET30 (Novagen) as a template, by removing 117 bp 3' of the histidine tag site that encoded extra affinity tags and adding the sequence 5'TCCGGTATTGAGGGTCGCTCTAACT CTCCTCTG- 3' to allow for Ligation Independent Cloning (LIC). Following linearization within the LIC sequence (BseR1) and blunt-ending with mung bean nuclease, LIC cloning was performed using existing protocols (Novagen). Cells were grown under different expression conditions (host cell and media type, temperature, induction conentration, and time). Two µl of cell lysate were spotted onto Protran membrane. Immunoblotting using penta-His primary antibody (Qiagen), HRP goat antimouse secondary antibody (Jackson Immunoresearch) and DAB substrate (Pierce) determined the level of total and soluble protein. Proteins were then expressed in 5 ml culture volumes in 24 well blocks. Cleared cell lysates were then batch loaded in a Ni-NTA superflow resin (Qiagen) in a 96-well filter plate (4°C, 20 min), and the column was formed by applying vacuum. Following three 750 µl washes, the protein was eluted by adding 100 µl elution buffer (containing 250 mM imidazole) followed by applying vacuum. Protein quantity and quality are determined using the Bioanalyzer Lab on a Chip (Agilent).

A. Expression Screen

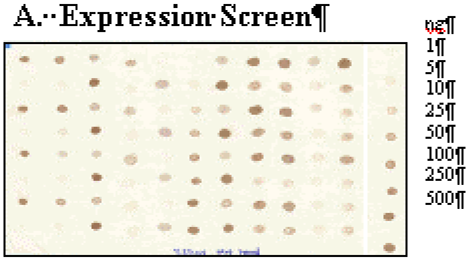

B. Protein Analysis

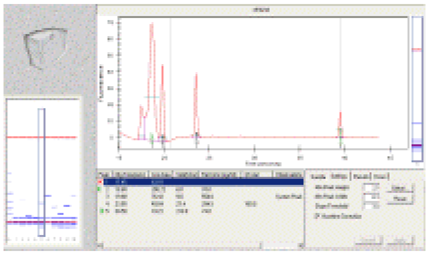

**RESULTS**. Our custom LIC vector allowed for fast and efficient subcloning, adding only six histidines and six additional residues (SGIEGR) at the N-terminus of the protein sequence. The expression screen identified optimal conditions for production of protein, with a sensitivity of 5–10 ng. Protein purification in 96-well format quickly produces up to 200 ng pure protein. Quality and yield are accurately determined using the Agilent Bioanalyzer. These methods allowed for an overall success rate of 70%.

**DISCUSSION.** Utilizing an efficient cloning method such as LIC with a protein expression screen enables the identification of optimal conditions for protein production. In addition to our N-terminal histidine tag vector, other vector tags (C-terminal histidine and fusion protein tags) are also amenable to the LIC system. Modifications of standard Ni-NTA protein purification protocols allow for increased yield and purity of proteins.

**ACKNOWLEDGEMENTS**. This work was performed under the auspices of the U.S. Department of Energy, Office of Biological and Environmental Research, by the University of California, Lawrence Livermore National Laboratory (No. W-7405-Eng-48), Lawrence Berkeley National Laboratory (No. DE-AC03-76SF00098), and Los Alamos National Laboratory (No. W-7405-ENG-36).

**REFERENCE**

1. Aslanidis, C. and de Jong, P.J. (1990) Nucl. Acids Res. 18, 6069–6074.
